# Prospective Preference Assessment of Potential Participants in the Perioperative Rectal Methadone in Spine Surgery (PROMISE) Trial

**DOI:** 10.1080/24740527.2026.2717112

**Published:** 2026-10-01

**Authors:** Victoria Tucci, Mindy Lu, Nandana Parakh, Janneth Pazmino-Canizares, Roshni Nayar, Zaaria Thomas, Gabriella Mattina, Christopher D. Witiw, Jefferson Wilson, Karim Ladha, Duminda Wijeysundera, Akash Goel, Sérgio Martins Pereira

**Affiliations:** a Temerty Faculty of Medicine, University of Toronto, Toronto, Canada; b Department of Anesthesia, St Michael’s Hospital, Toronto, Canada; c Li Ka Shing Knowledge Institute, St Michael’s Hospital, Unity Health Toronto, Toronto, Canada; d Division of Neurosurgery, Department of Surgery, University of Toronto, Toronto, Canada; e Department of Anesthesiology and Pain Medicine, University of Toronto, Toronto, Canada

**Keywords:** Clinical trial design, perioperative analgesia, perioperative pain management, postoperative pain, prospective preference assessment, rectal methadone, spine surgery

## Abstract

**Background/Aims:**

Perioperative pain management in spine surgery is challenging, with many patients suffering from chronic pain. The favorable pharmacokinetic–dynamic profile of methadone may offer a novel approach to perioperative analgesia; however, stigma around methadone and rectal administration may bias patient enrollment. Before initiating a trial comparing rectal methadone to placebo, we conducted a prospective preference assessment (PPA) to identify barriers and motivators for participation and characterize differences between willing and unwilling participants to refine the trial protocol.

**Methods:**

Adult patients undergoing elective spinal surgery were recruited from St. Michael’s Hospital’s preoperative clinic from July to August 2024. Participants completed a PPA, including a trial vignette, assessment of understanding, open-ended questions exploring attitudes toward the trial, and a questionnaire. Data were analyzed with thematic analysis, t-test, and Fisher’s exact test.

**Results:**

Thirty participants were enrolled and categorized based on a 6-point Likert scale into “willing” to participate (Likert 4–6, 83.3%, CI 0.69–0.98) and “not willing” (Likert 1–3, 16.7%, CI 0.0–0.49). Pain as a major concern was significantly associated with willingness to participate, with 88% of willing participants reporting it as a concern compared to 40% of non-willing participants (p = .04). Common motivators included optimizing pain management (57%) and perceived benefits of rectal methadone (30%). Common concerns were methadone side effects (13%) and dependency fears (13%).

**Conclusion:**

We identified a strong willingness (83.3%, CI 0.69–0.98) among spine surgery patients to participate in our trial. Protocol adjustments will focus on patient education regarding methadone’s safety profile, clarifying measures to minimize dependency risks, and ensuring physiologic monitoring.

## Résumé

**Contexte/Objectifs :** La prise en charge de la douleur périopératoire en chirurgie rachidienne est complexe, notamment parce que de nombreux patients présentent une douleur chronique. Le profil pharmacocinétique et pharmacodynamique favorable de la méthadone pourrait offrir une nouvelle approche de l’analgésie périopératoire. Toutefois, la stigmatisation associée tant à la méthadone qu’à son administration par voie rectale pourrait influer sur le recrutement des patients. Avant d’entreprendre un essai comparant la méthadone administrée par voie rectale à un placebo, nous avons mené une évaluation prospective des préférences. Celle-ci visait à cerner les obstacles et les facteurs favorisant la participation ainsi qu’à caractériser les différences entre les personnes disposées à participer et celles qui ne l’étaient pas, afin d’affiner le protocole de l’essai.

**Méthodes :** Des patients adultes devant subir une chirurgie rachidienne programmée ont été recrutés à la clinique préopératoire de l’Hôpital St. Michael’s entre juillet et août 2024. Les participants ont pris part à une évaluation prospective des préférences comprenant une vignette décrivant l’essai, une évaluation de leur compréhension, des questions ouvertes explorant leurs attitudes à l’égard de l’essai ainsi qu’un questionnaire. Les données ont été analysées au moyen d’une analyse thématique, d’un test t et du test exact de Fisher.

**Résultats :** Trente participants ont été recrutés et répartis, selon leurs réponses à une échelle de Likert à six points, entre les catégories « disposés à participer » (scores de 4 à 6, 83,3 %; IC : 0,69–0,98) et « non disposés à participer » (scores de 1 à 3, 16,7 %; IC : 0,00–0,49). Le fait de considérer la douleur comme une préoccupation majeure était significativement associé à la volonté de participer : 88 % des participants disposés à participer ont déclaré qu’elle constituait une préoccupation, comparativement à 40 % de ceux qui ne l’étaient pas (p = 0,04). Les principaux facteurs favorisant la participation étaient l’optimisation de la prise en charge de la douleur (57 %) et les bienfaits perçus de la méthadone administrée par voie rectale (30 %). Les principales préoccupations concernaient les effets indésirables de la méthadone (13 %) et le risque de dépendance (13 %).

**Conclusions :** Nous avons constaté une forte disposition à participer à notre essai (83,3 %; IC : 0,69–0,98) chez les patients devant subir une chirurgie rachidienne. Les ajustements apportés au protocole porteront sur les renseignements fournis aux patients concernant le profil d’innocuité de la méthadone, la clarification des mesures visant à réduire au minimum le risque de dépendance et la mise en place d’une surveillance physiologique.

## Introduction

Perioperative pain management in spine surgery is challenging, with approximately 15% of postoperative patients experiencing chronic pain.^1^ Surgical trauma and tissue injury associated with a history of chronic pain and preoperative opioid use place these patients at risk for suboptimal pain management and related complications.^2,3^ Traditionally, postoperative pain management has consisted of intermittent administration of shorter-acting opioids, such as morphine and hydromorphone.^4^ Yet, this strategy can produce fluctuating blood opioid concentrations, leading to clinical responses that may range from respiratory depression to inadequate pain management.^4^ Ketamine, an NMDA-receptor antagonist, is incorporated into North American and European guidelines for complex spine surgery; however, its perioperative use is often constrained by the need for continuous intravenous infusion and titration, dose-dependent psychotomimetic and dissociative effects, and requirements for enhanced monitoring.^5,6^ Alternative strategies, such as nerve blocks, lidocaine, and dexmedetomidine, lack consensus.^5,7^ As a result, there is a clear and unmet need for novel pain-management approaches for patients undergoing spine surgery.

Methadone, a long-acting μ-opioid agonist and NMDA antagonist, offers distinct perioperative advantages, as it serves as a single-drug multimodal analgesic by binding to multiple receptors.^8^ Its prolonged half-life ensures steady plasma levels, reducing acute pain escalation, opioid use, and chronic pain.^9–12^ Additionally, its NMDA antagonism attenuates central sensitization, potentially preventing the transition from acute to chronic postsurgical pain, making it a promising option for spine surgery.^9–12^ In Canada, methadone is only indicated for oral administration; however, this route can be limited in the perioperative setting by restricted intake and gastrointestinal dysfunction, which may delay or destabilize absorption when timely analgesia is needed.^13,14^ Conversely, rectal administration provides a nonparenteral alternative in these circumstances and demonstrates a favorable pharmacokinetic profile, achieving reliable drug absorption and therapeutic plasma levels.^12^ Pharmacokinetic data in healthy volunteers show that rectal dosing with an aqueous methadone solution achieves approximately 80% relative bioavailability (AUC–8 h) compared with oral, with a similar time to peak concentration (T_max_ 2.8 vs 3.1 h), supporting rectal methadone as a feasible strategy to maintain systemic exposure when oral administration is suboptimal.^12,15–17^ Despite these advantages, rectal methadone has not yet been studied for perioperative pain management.

To address this gap, we propose the PeRiOperative Rectal Methadone In Spine SurgEry (PROMISE) Study (clinicaltrials.gov: NCT06843174), a randomized, placebo-controlled trial in participants undergoing elective spinal surgery to evaluate the feasibility of incorporating rectal methadone into the perioperative pain management protocol. Prior to initiating the PROMISE Study, we conducted a prospective patient preference assessment (PPA) to proactively address common challenges that pose barriers to the efficiency, generalizability, and success of clinical trials, i.e., inadequate and selective enrollment and participant non-adherence.^18^ Several features of the PROMISE intervention may pose particular barriers to recruitment. Methadone carries substantial societal stigma owing to its established role in the treatment of opioid use disorder, and patients frequently report fears of dependency and addiction associated with opioid-based analgesia.^19,20^ In addition, the rectal route of administration is uncommon in contemporary perioperative practice and may be perceived as being unfamiliar. Because these perceptions could deter participation and bias enrollment toward patients with more favorable attitudes, a PPA was warranted to characterize these barriers before trial initiation and to inform protocol refinements that maximize recruitment. The primary objectives of this PPA are as follows: (1) to identify patient concerns and motivations regarding participation in the PROMISE Study; (2) to pinpoint areas in the trial protocol requiring modification to enhance enrollment and adherence; (3) to assess differences in characteristics between patients willing and unwilling to participate.

## Methodology

### Setting and population

This research was approved by the Unity Health Toronto Research Ethics Board (approval #24-135 on 07/20/2024). Written informed consent was obtained from all participants, who were also informed of their right to withdraw from the study at any time. Patients who had in-person appointments at the Preadmission Facility (PAF) during the recruitment period were screened for eligibility based on the procedure type indicated on the PAF patient list. A purposive sampling framework^21^ was applied to recruit patients scheduled for elective spinal fusion surgery at St. Michael’s Hospital (SMH), Toronto, Canada, from July to August 2024, as this is the target population for the planned PROMISE trial and the center where the PROMISE trial will be initiated. Eligible patients were approached in-person for consent before or after their preoperative appointment. They received a brief overview of the PPA design and general methodology of the proposed PROMISE trial in the Letter of Information and Consent to Participate forms.

Adult patients (≥18 years old) were included if they were scheduled for elective spinal fusion of one or more sacral, lumbar, thoracic, and/or cervical levels and had the capacity to consent to the research study. Patients were excluded if they did not meet these criteria and/or did not have a working knowledge of English.

This study aimed for a target sample size of 35 participants, aligning with the typical range of 20 to 50 participants in previously published PPAs.^22,23^ A moderate sample size of 35 was selected to ensure adequate demographic and clinical diversity among participants to support the validity and generalizability of the PPA findings. Data collection was guided by the principle of saturation, with recruitment concluding when interviews ceased to provide additional insights.

### PPA survey design

A four-phase, 15-min PPA survey was conducted in person with each consenting patient to capture their perspectives and characteristics. The first phase provided a brief vignette outlining the proposed PROMISE trial, including the rectal methadone intervention, randomization, blinding, and data collection. The second phase assessed participants’ understanding of the trial through three interviewer-administered questions about the trial’s structure. The third phase involved open-ended interview questions exploring attitudes toward participation and influencing factors, with responses securely recorded on an encrypted audio recorder and transcribed verbatim by the interviewer (VT). The final phase consisted of a self-administered questionnaire evaluating willingness to participate via a 6-point Likert scale by asking, “Considering this information, how willing would you be to participate in this study if it were to happen in the future.?” Options included 1 = definitely not; 2 = probably not; 3 = maybe not; 4 = maybe; 5 = probably; and 6 = definitely. The questionnaire also assessed 13 baseline patient characteristics, including pain medication use and frequency of pain (Appendix I). To minimize social desirability bias, patients completed the questionnaire privately.^24^

### Quantitative and qualitative data analysis

Quantitative analysis included mean, standard deviation, and 95% confidence interval (CI) estimations for continuous variables (with normality confirmed by the Shapiro–Wilk test, p = .169), and percentages for categorical variables, including willingness to participate. The “willingness to participate” variable was measured using a 6-point Likert scale ranging from “definitely not” (1) to “definitely” (6) and, for the primary feasibility analysis, was dichotomized into “not willing” (Likert 1–3) and “willing” (Likert 4–6). This threshold reflects the internal structure and directional framing of the response scale. The lower three responses (“definitely not,” “probably not,” and “maybe not”) indicate an overall negative orientation toward participation, whereas the upper three responses (“maybe,” “probably,” and “definitely”) indicate varying degrees of openness to participation.^25,26^ Thus, the boundary between Likert responses of 3 and 4 distinguishes participants with reluctance toward enrollment from those expressing at least conditional openness. This approach also reflects the practical purpose of the PPA, which was to determine whether patients may be open to enrollment if modifiable concerns are addressed through education, consent discussions, and protocol clarification. A response of “maybe” (Likert 4) was therefore classified as reflecting conditional openness to enrollment rather than refusal. This dichotomization is consistent with our group’s prior PPA conducted for the CARES trial,^23^ in which the same instrument and thresholds were applied. Means and percentages for patient characteristics were stratified accordingly. Between-group differences were assessed using t-tests for continuous variables and Fisher’s Exact Test for categorical variables.

Qualitative data were analyzed via thematic analysis using the MAXQDA software (Version 24.7.0. Berlin: VERBI Software; 2021) to assess emerging patterns, codify, and extract meaning from de-identified participant interview data through both inductive and deductive interpretations to identify factors that motivate or discourage participation in the PROMISE trial.^27–29^ Two independent researchers (VT, ML) coded each transcript for consistency, with disagreements resolved by the principal investigator (SMP). The final transcripts incorporated agreed-upon codes.

Transcripts were thoroughly reviewed for familiarity before generating initial codes to capture recurring ideas. These codes, representing meaningful concepts expressed by participants, were systematically applied and organized into broader categories of motivators and discouraging factors.^30^ Themes were refined iteratively to ensure accurate representation. A codebook – including code names, definitions, examples, and analytic summaries – was developed and used for a second coding round to ensure consistency.^31^ To maintain accuracy, repetitive mentions from the same participant were counted only once, ensuring code frequency reflected the number of participants expressing a concept rather than total mentions. Similar concepts were consolidated into overarching themes related to the willingness to participate in the PROMISE trial.

## Results

### Patient and clinical characteristics of the sample population

Overall, 751 patients were preliminarily screened for eligibility by reviewing their procedure type indicated on the PAF patient list. Of these, 47 patients met the inclusion criteria and were approached and assessed for eligibility (Figure 1). After reviewing the informed consent form, 17 patients declined enrollment for reasons including time constraints (n = 2), early departure (n = 1), lack of interest (n = 1), not comfortable elaborating in English unassisted (n = 1), psychological concerns (n = 2), discomfort with study methodology (n = 1), personal reasons (n = 5), planning to refuse surgery (reasons unrelated to potential trial involvement) (n = 2), or uncertainty about surgery (n = 2). Ultimately, 30 participants were enrolled and completed the PPA.

**Figure 1.**
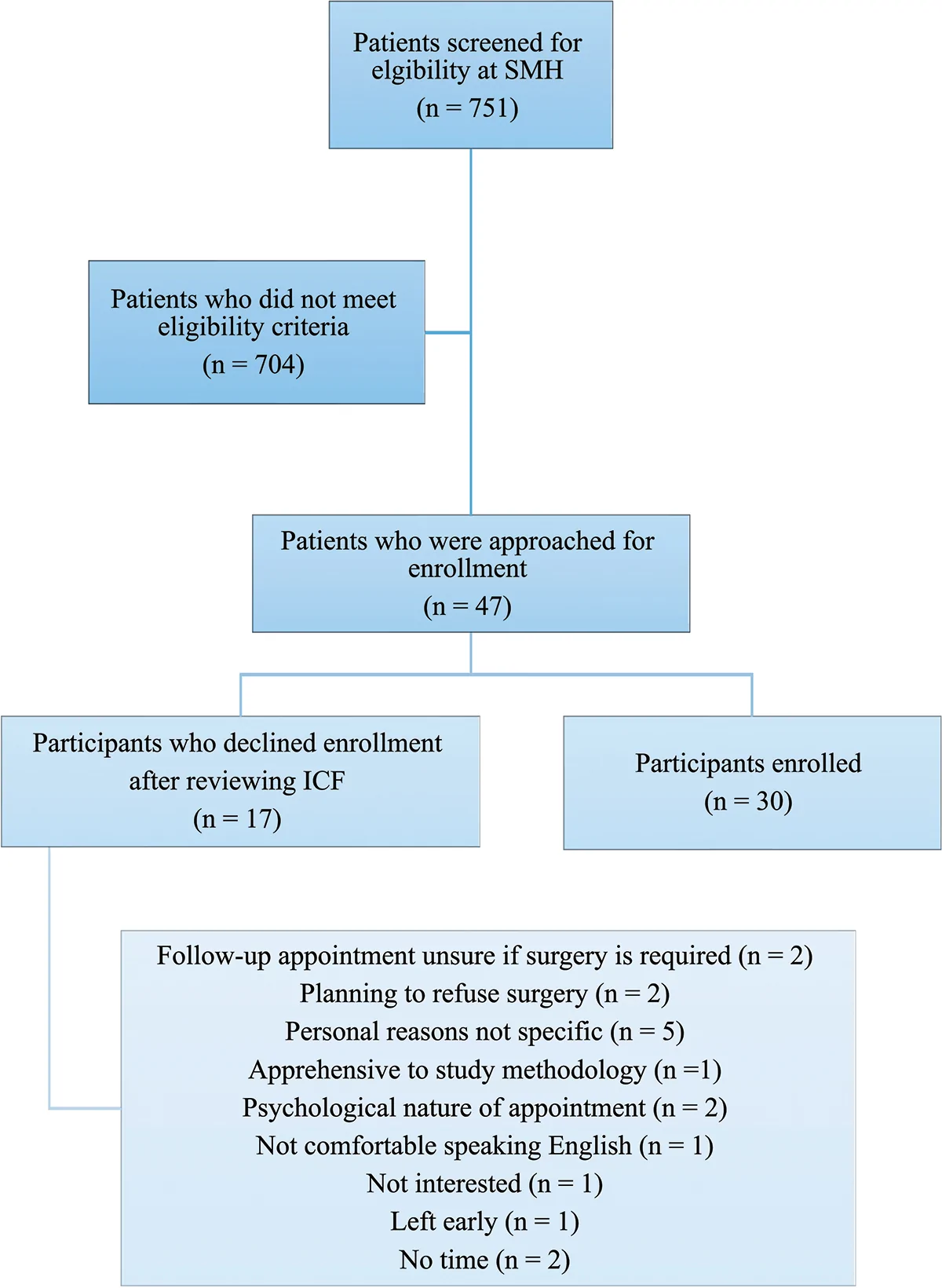
Patient enrollment flow chart.

Participants had a mean age of 66.8 years (SD 11.4, range 33–89), with the majority being male (60%), defined by sex assigned at birth. Eighty percent identified as White/Caucasian, and 57% had at least 15 years of education, though only 17% had prior research experience. Most participants (n = 25, 83.3%, CI 0.69–0.98) were willing to participate in the PROMISE trial, with 33% “definitely” and 27% “probably” willing, while a minority were “definitely not” (7%) or “probably not” (7%) interested (Table 1).

**Table 1.** Characteristics of participants who were not willing (n = 5) and willing (n = 25) to participate in the PPA-PROMISE trial.

	Total (n = 30), N (%) or mean (SD)	Not willing to participate (n = 5, 16.7%) N (%) or mean (SD)	Willing to participate (n = 25, 83.3%) N (%) or mean (SD)
**PATIENT CHARACTERISTICS**			
Age (years)	66.8 (11.4)	67.4 (10.3)	66.7 (11.8)
**Sex assigned at birth**			
Female	12 (40)	2 (40)	10 (40)
Male	18 (60)	3 (60)	15 (60)
**Visible Minority**			
Yes	6 (20)	0 (0)	6 (24)
No	24 (80)	5 (100)	19 (76)
**Years of School Completed**			
≥15	17 (57)	2 (40)	15 (60)
<15	13 (43)	3 (60)	10 (40)
**Participated in a research study before**			
Yes	5 (17)	0 (0)	5 (20)
No	22 (73)	5 (100)	17 (68)
Not sure	3 (10)	0 (0)	3 (12)
**If yes, what is your status?**			
No response	26 (87)	5 (100)	21 (84)
Completed the study	3 (10)	0 (0)	3 (12)
Still an active participant (on-going study)	1 (3)	0 (0)	1 (4)
**CLINICAL CHARACTERISTICS**			
**Concomitant medical conditions**			
Yes	15 (50)	1 (20)	14 (56)
No	15 (50)	4 (80)	11 (44)
**Prior surgery**			
Yes	28 (93)	5 (100)	23 (92)
No	2 (7)	0 (0)	2 (8)
**Currently consuming medication for pain**			
Yes	21 (70)	3 (60)	18 (72)
No	9 (30)	2 (40)	7 (28)
Pain as a major concern *			
Yes	24 (80)	2 (40)	22 (88)
No	6 (20)	3 (60)	3 (12)
**Previous discussions regarding post-operative pain management**			
Yes	10 (33)	1 (20)	9 (36)
No	20 (67)	4 (80)	16 (64)
**Significant pain on most days over the past 3 months**			
Yes	20 (67)	4 (80)	16 (64)
No	10 (33)	1 (20)	9 (36)
**If yes, are you managing your pain well?**			
No response	8 (27)	1 (20)	7 (28)
Yes	14 (47)	3 (60)	11 (44)
No	8 (27)	1 (20)	7 (28)
**Use of medication for pain for ≥1 week over the last month**			
Yes	19 (63)	4 (80)	15 (60)
No	11 (37)	1 (20)	10 (40)
**Use of anything other than medication to manage pain for ≥1 week over the last month**			
Yes	20 (67)	3 (60)	17 (68)
No	10 (33)	2 (40)	8 (32)

* Indicates a statistically significant difference between groups; Data presented as N (%) or mean ± SD.

### Participant characteristics influencing willingness to participate

Willingness responses spanned the full 6-point scale: 10 participants (33.3%) were “definitely” willing, 8 (26.7%) “probably,” 7 (23.3%) “maybe,” 1 (3.3%) “maybe not,” 2 (6.7%) “probably not,” and 2 (6.7%) “definitely not.” When these responses were dichotomized, most participants were classified as “willing” (Likert 4–6; n = 25, 83.3%, CI 0.69–0.98) and a minority as “not willing” (Likert 1–3; n = 5, 16.7%, CI 0.0–0.49). The difference in willingness to participate between groups was assessed using Cohen’s h, which yielded a large effect size of 1.44 (CI 0.48–2.4). The only significant between-group difference was in “pain as a major concern regarding spine surgery” (p = .04), with those reporting pain as a major concern being more likely to participate (88% vs. 40%, p = .04). No significant differences were found for other factors, including age, sex, education, visible minority status, prior research participation, medical conditions, surgical history, pain medication use, or previous pain discussions. While not statistically significant, potential factors influencing willingness included ≥15 years of education (60% vs. 40%), prior research participation (20% vs. 0%), and concomitant medical conditions (56% vs. 20%) (Table 1).

### Motivating factors and concerns influencing willingness to participate

Among the 25 independent themes identified from 30 participant interviews, 10 key themes were found to relate to factors motivating participation in the PROMISE Trial (Table 2), and 15 themes were identified that relate to factors discouraging participation (Table 3). Figures 2, 3 displays theme frequency, delineated by willingness to participate. The most reported motivator for participation was optimizing pain management (57%), followed by the perceived benefit of rectal methadone (30%). Even those unwilling to participate cited pain relief, methadone benefits, and comfort with rectal administration as motivating factors. Altruism was commonly mentioned as a motivating theme among 27% of the total participants and 36% among patients willing to participate. Similarly, scientific and knowledge advancement was mentioned as a motivating theme among 23% of the total participants and 28% among patients willing to participate. Of the 59 total responses for motivating themes, a greater proportion was derived from the willing to participate group (95%). Several motivating themes were unique to this group and reported only by willing participants included curiosity (24%), familiarity with methadone (8%), scientific/knowledge advancement (28%), altruism (32%), minimizing postoperative opioid use (12%), and ambivalence (4%) (Figure 2).

**Figure 2.**
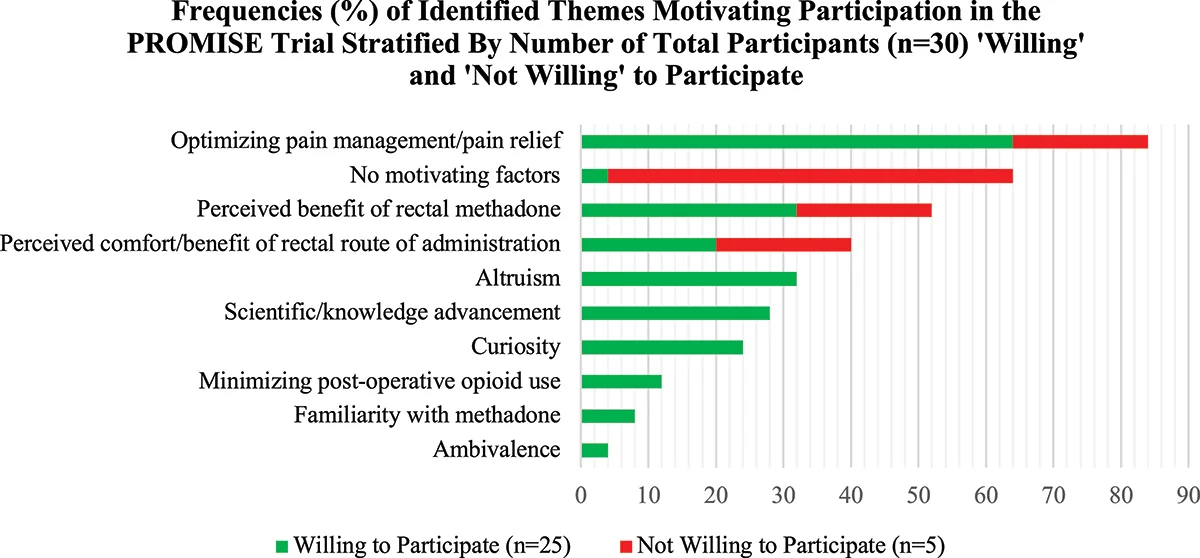
Frequencies of themes motivating participation in the PROMISE Trial, categorized by participants’ willingness to engage (‘willing’ vs. ‘not willing’).

**Table 2.** Key themes and quotes identified from patient interviews regarding factors that motivate participation in the PPA PROMISE trial.

Factors Motivating Participation		
Identified Themes	Descriptive Note	Key Patient Quotes
Optimizing pain management/pain relief	Pain relief emerged as a primary motivator for participation, with many participants drawn to the prospect of improved pain control while minimizing reliance on opioids. The opportunity to proactively address anticipated post-surgical or chronic pain resonated strongly, particularly among those expecting significant discomfort after surgery.	“To help with the pain” not willing, female, 63 “To avoid pain,” willing, male, 75 “The pain … to remove some of the pain,” willing, male, 71 “I think that if it’s pain management and if pain management can be quickened then ya absolutely, I think it’s a great idea,” willing, male, 61 “Definitely not feeling any pain um getting the aftereffects and waking up and […] feeling a little bit better than perhaps the pain that could ensue later,” willing, male, 33 “No pain, I would do it to help with pain,” willing, female, 76
Perceived benefit of/comfort with rectal route of administration (independent of methadone)	Some participants directly expressed comfort with the rectal administration method (independent of methadone being the administered drug), appreciating its benefits and showing openness to innovative delivery mechanisms. Those with prior knowledge or comfort with such methods tended to view it positively.	“Well, the whole rectal thing doesn’t scare me” willing, male, 61 “From a rectal standpoint, I understand the absorption, I think it’s a good idea, I don’t see any issues. If you’re under general anesthesia you’re not gonna feel anything anyway so from that standpoint I think it’s a great idea,” not willing, male, 56 “I’m willing- the methadone isn’t gonna do me any harm even rectally- it just sounds so gross, but you know what people have to get suppositories so it’s the same thing, it’s all just part of the anatomy so it’s not a big deal when you look at it scientifically,” willing, female, 63
Perceived benefit of rectal methadone	Some participants shared their belief in, and understanding of, the potential benefits of rectal methadone especially in comparison to other pain management methods. They viewed it as a promising option for reducing opioid use and improving post-surgical pain relief, particularly if it could provide longer-lasting pain control.	“Well, we know methadone really is a really good drug ‘cause it you know kinda helps you get off heroin, cocaine that sorta thing. Having done heroin in my life, not an extended period of time but trying heroin, I can see that methadone may be the way to go,” willing, male, 61 “It’s gonna be rectal right, and it’s supposed to be when I’m done at the surgery gradually the pain will not be bad, okay that’s what I’d like,” willing, male, 71 “If that’s the best way to help with pain then that’s fine and would be good with that” willing, male, 48
Curiosity	Participants expressed curiosity about the trial, including its methodology, the drug’s mechanism, or the ability to achieve better pain relief. For some, this intrinsic interest was a sufficient motivator for participation.	“Just curiosity I guess,” willing, female, 75 “Should my pain increase and if that happens then I would be interested in finding out what more I could do,” willing, male 75
Familiarity with methadone	Participants who were familiar with methadone, whether through personal use, medical knowledge, or prior exposure, often displayed more interest in the trial. Familiarity bred a sense of confidence or curiosity about the drug’s potential application in pain management. For some participants, currently consuming opioids made them more comfortable with the idea of participating in the study, as they were already familiar with opioid-based pain management. This familiarity provided a sense of ease and confidence in the potential benefits of the trial.	“I would know how much it would help me the rectal methadone,” willing, female, 72 “Well, we know methadone really is a really good drug ‘cause it you know kinda helps you get off heroin, cocaine that sorta thing,” willing, male, 61
Scientific/ **knowledge advancement**	Participants were inspired by the opportunity to contribute to scientific advancements, often framing their participation as a way to help expand medical knowledge. Many acknowledged the importance of clinical trials in discovering new pain management solutions. Participants valued the trial’s potential to drive innovation, even when they lacked direct personal benefits from the research.	“Well, anything for research, I would participate in it for sure,” willing, female, 52 “If it helps if it helps people with pain then you know the only way to finding out is by trial,” willing, male, 68 “To help with the research,” willing, male, 62 “Just the research factor,” willing, male, 67 “I’m all for research,” willing, male, 75 “To help with pain and research for other patients in the future,” willing, male, 48
Altruism	Participants were motivated by the desire to help others and contribute to research that could benefit future patients. Many framed their involvement as an act of goodwill, citing satisfaction in knowing their participation could improve outcomes for others undergoing similar challenges with chronic and/or anticipated post-surgical pain. Altruism appeared frequently in conjunction with research motivations, reflecting a blend of patient interest in societal and scientific contributions.	“To help future patients,” willing, female, 79 “So that maybe other people have better benefits later on,” willing, female, 63 “The chances of becoming addicted to opioids is high so um anything we can do to mitigate that for patients is wonderful, I’m happy to be participating,” willing, male, 67 “Just helping other people,” willing “If I could do something to help patients in the future that’s good,” willing, female, 74 “To help with pain and research for other patients in the future,” willing, male, 48 “To avoid pain and to help others to avoid pain,” willing, male, 75
Minimizing post-operative opioid use	Participants were motivated by the potential to decrease dependency on stronger and more addictive medications for post-surgical pain. They recognized that rectal methadone might help reduce the need for traditional opioids, aligning with broader goals of minimizing opioid dependence and promoting safer pain management practices. This was especially salient for those with personal or professional awareness of opioid risks, those seeking alternatives to stronger, more addictive pain medications, or those who had negative past experiences with opioids.	“To get off opioids,” willing, female, 83 “Reducing the amount of opioids, you would have to take to manage the pain afterwards,” willing, male, 64 “Chances of becoming addicted to opioids is high so um anything we can do to mitigate that for patients is wonderful,” willing, male, 67
Ambivalence	A participant displayed ambivalence, expressing uncertainty about the trial or the intervention, with mixed feelings about the potential benefits and risks.	“Um so right now like it’s premature right so ya like if we have fully mature then we can do that […] but right now ya we are just thinking of that […] so it’s not sure,” willing, male, 62
No motivating factors	Some participants reported no strong motivating factors for participating, often expressing skepticism, disinterest, or ambivalence toward the study.	“None at this time,” not willing, male, 65 “None, I don’t believe in any pain medication at all,” not willing, male, 80

**Table 3.** Key themes and quotes identified from patient interviews regarding concerns with participation in the PPA-PROMISE trial.

Factors Discouraging Participation		
Identified Themes	Descriptive Note	Key Patient Quotes
Concerns about long-term methadone use and effectiveness	A participant expressed concerns about the long-term benefits of the intervention for pain relief, specifically questioning its efficacy in managing pain beyond the trial period and the frequency of its required use for sustained benefit.	“I guess it would be around the safety of using it and how often you would have to use it after the fact and to the degree its gonna help support your pain like how effective is it,” willing, male, 57
Safety of methadone	A participant raised concerns about the safety of methadone, particularly in its role as both an opioid and a treatment for opioid dependence. While acknowledging its use in recovery, they expressed apprehension about its long-term safety profile.	“Well methadone is an opioid right so there’s clearly concerns about taking opioids but I do think they use opioids to help people get off other opioids so I guess it would be around the safety of using it,” willing, male, 57
Side effects of methadone	Some participants were specifically concerned about the physical and/or psychological side effects of methadone, whether in general or during their surgery, with particular concerns about the drug altering their state of mind or physiology.	“I think it’s just the risk […] just not necessarily knowing what the potential side effects, if any, can be,” willing, male, 33 “The side effects,” willing, female, 76
Opposition to receiving placebo	Some participants preferred to have assurance that they would receive the methadone rather than placebo. This concern was often rooted in the belief that receiving a placebo is less likely to benefit them or alleviate their pain.	“I do not want the placebo. I do not want anything put in my anus that is not gonna be beneficial for me,” willing, female, 63
Stigma around methadone	Some participants shared concerns about the negative societal perceptions of methadone, often linked to its use in opioid substitution therapy. Participants may hesitate to use methadone due to its association with addiction treatment, fearing judgment or stigma.	“The methadone isn’t a positive for me just be the nature of the drug itself and the ramifications and that sort of thing,” willing, male, 61 “Just the stigma around methadone right, I think that’s my big one it’s never had a good rep because of what it does and it’s a lifelong- I work in the mental health industry believe it or not and it’s not recovery it’s bandaid so I think that would be the only thing,” willing, male, 61 “Well methadone is an opioid right so there’s clearly concerns about taking opioids,” willing, male, 57
Influence on perception of pain	A participant was concerned that the administration of methadone before surgery may alter their perception of pain, potentially influencing their pain management decisions. They questioned whether the medication could interfere with their ability to gauge the intensity of pain during recovery. This reflects a concern that the drug’s effects might cloud their natural pain thresholds, potentially hindering timely interventions.	“Does it mask any kind of your perceptions of your pain? Obviously, you know how you’re supposed to control pain at a constant level right so you don’t wait ‘til it gets really terrible before you administer it but does by receiving it before kind of mask some of that so you could be late for that?,” willing, male, 62
Impact on post-surgical pain management	A participant expressed concerns about how the administration of methadone during surgery might affect their post-operative pain management. Specifically, despite clarification and reassurance, they questioned whether receiving methadone would influence the type and dosage of pain relief offered after the procedure.	“I guess my only concern is how does that affect what you’re given post-surgery because if you don’t know what you’ve gotten before?,” willing, male, 62
Impact on surgical performance, outcomes and/or timeline	Some participants had concerns related to the potential effects of rectal methadone on the surgical procedure itself, including its impact on the performance of the surgical team, the expected outcomes, or the surgery timeline. Participants worried that receiving a treatment prior to surgery could alter their physiological responses or interfere with the surgical process, leading to unintended complications or delays.	“Sometimes something can be discovered during the study that prevents you from having the procedure,” willing, female, 79 “Does it affect the body’s response during the surgical operation? […] Will it affect what the surgeon does? […] Ok, I just need to know that it won’t affect the operational performance […] I get they’re blinded but since I have this medication in my body does it affect the surgical procedure at all? […] Does that have any sort of physiological response to the surgical procedure and in that process does it tweak something or surprise the surgeon while he’s doing his thing so that would be my only concern,” willing, male, 62
Skepticism/hesitance toward analgesics in general	Participants who exhibit skepticism or hesitance toward analgesics in general, including opioids and alternative methods, often express a preference for managing pain through non-pharmacological means. This hesitation may stem from past experiences with medication, concerns about dependency, or a belief in the body’s natural ability to handle pain.	“I would just like to minimize the amount of pain medication that I take,” not willing, male, 65 “I prefer not to take it if I don’t have to,” willing, male, 57 “I don’t believe in any pain medication at all. I don’t like pain medications, and I feel that 90% of those people should be able to handle that pain within their body and it’s all mind control. I’ve suffered with pain since 1982 with a back injury and I don’t take anything other than a couple of Tylenol once in a while and I’ve managed it and I’ve learned to live with it,” not willing, male, 80
Fear of potential for dependency or addiction	Concerns about the potential for addiction or dependency on opioids, including methadone, were prevalent. Many expressed fear that using opioids could lead to long-term reliance. These concerns were particularly emphasized by those with prior experiences with pain medications or those familiar with the risks of opioid use.	“The use of opioids, due to their addictive nature,” willing, male, 48 “So, when it comes to opiates, I’m very nervous. I’m also a chemistry teacher so when it comes to opiates and heavier pain meds I try to stay away from them. And I’d rather go through the pain than be on the opiates because I’m scared of the dependency and all the other factors that occur,” not willing, male, 56 “I’ve seen too many people taking the pain medication and they rely on it, they rely on it continuously,” not willing, male, 80 “I am very um the pain I can handle it but I don’t want to take any addictive medication my pain tolerance too high so I’ve been doing fine but still I can handle it,” willing, male, 71
Blinding to intervention assignment	A participant expressed concern about not knowing which treatment they will receive, indicating discomfort with the uncertainty of randomization.	“That is a concern then if I don’t know and I just get this IV, I think I should know ahead of time so I can prepare it in my mind,” willing, female, 72
Novelty of study	Some participants shared concerns about the early stage of the research. This often resulted in hesitation or uncertainty, as some participants expressed a desire for more robust evidence or proof of effectiveness before committing to the trial. While the potential benefits of the novel approach were recognized, participants conveyed a natural cautiousness, seeking reassurance about the safety and efficacy of the intervention.	“It’s premature like if it’s studied it more then we have more proof then yeah it’s good to go otherwise we can still think about it,” willing, male, 62 “I think it’s just the risk having it be a new study,” willing, male, 33
Lack of education/knowledge regarding study drugs	Participants expressed a desire for more education about the study drugs, particularly rectal methadone. The lack of knowledge led to more questions about the appropriateness of methadone use in this setting.	“I know I’ll be taking some pain medication probably after the surgery so I’m not sure whether taking methadone during the surgery is appropriate or not,” not willing, male, 65 “I think as a participant may be uh, they’d be looking for a little more education,” willing, male, 64
Ambivalence	Some participants displayed ambivalence, expressing uncertainty about the trial or the intervention, with mixed feelings about the potential benefits and risks.	“Um so right now like it’s premature right so ya like if we have fully mature then we can do that […] but right now ya we are just thinking of that […] so it’s not sure,” willing, male, 62
No concerns	Many participants expressed no concerns about participating in the trial.	“I don’t think there are really any concerns,” willing, male, 64 “I don’t even know if I have any concerns about it like I think it’s a great idea,” willing, female, 54 “Nope no concerns,” willing, female, 75 “None, if you were gonna say we’re gonna start next week I would come, this is great, I’m fine with that,” willing, male, 67 “None, I’m all for research and research of this nature is sure to be revolutionary so I don’t have any concerns. I’d be anxious to be a participant,” willing, male, 75 “Not much, it’s okay,” not willing, female, 63

**Figure 3.**
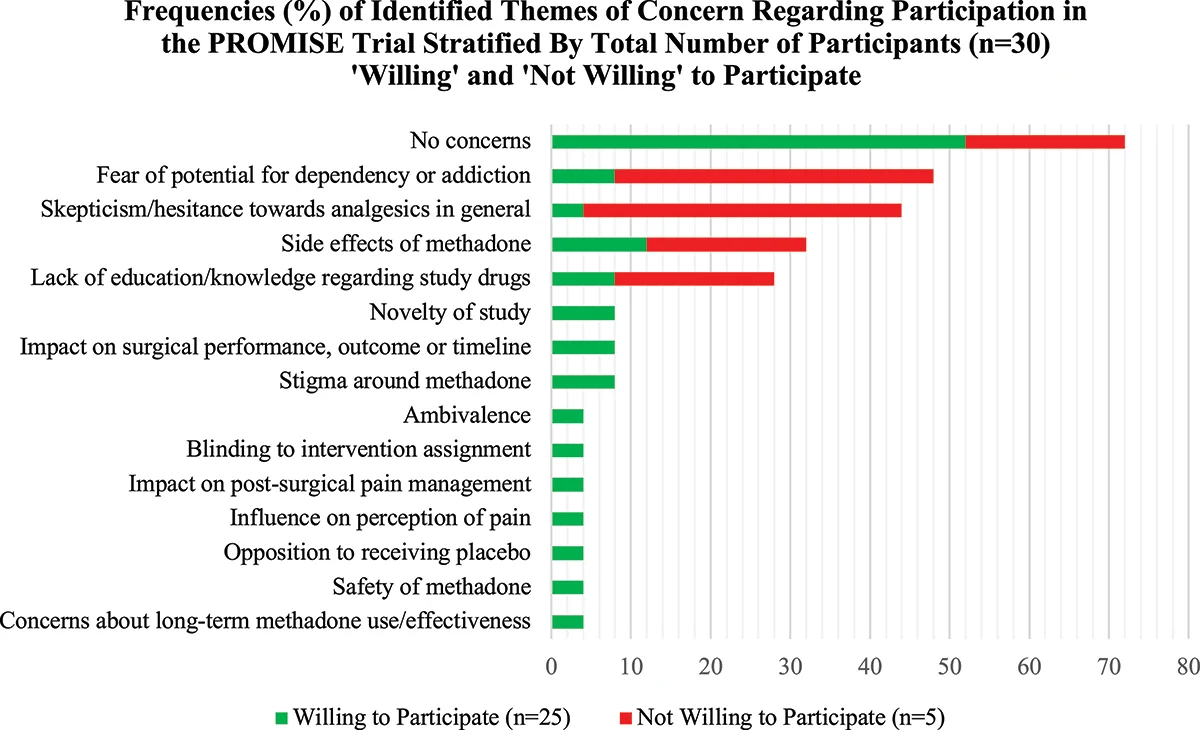
Frequencies of concerns related to participation in the PROMISE Trial, categorized by participants’ willingness to engage (‘willing’ vs. ‘not willing’).

The most common concerns discouraging participation were methadone side effects (13%) and fear of dependency/addiction (13%), followed by skepticism toward analgesics (10%) and lack of education on study drugs (10%). Among the five “not willing” participants, two cited addiction fears and skepticism toward analgesics. Four participants overall (60% from the “not willing” group) reported no motivating factors. Of 27 responses citing discouraging themes, 78% came from willing participants. Unique concerns reported only by willing participants included ambivalence (4%), study novelty (8%), blinding to intervention assignment (4%), impact on surgery/timeline (8%), post-surgical pain management (4%), pain perception (4%), methadone stigma (8%), placebo opposition (4%), methadone safety (4%), and concerns about long-term effectiveness (4%) (Figure 3). Despite these concerns, 78% of the participants remained willing to participate in the PROMISE trial. Overall, 47% of the participants had no concerns, with the majority in the willing group (52% vs. 20%), suggesting that willingness may not always correlate with explicit concerns (Appendix II).

## Discussion

This PPA study evaluated the willingness of patients undergoing elective spine surgery to participate in the PROMISE trial. Most of our participants expressed willingness to enroll, indicating strong acceptability for the proposed trial and high feasibility for recruitment. These findings suggest that the PROMISE study is well positioned for success in achieving its enrollment targets, highlighting the interest and openness among spine surgery patients toward innovative analgesic approaches.

### Key findings and implications

Willingness to participate in our study was high, with 83.3% of the participants indicating they would enroll. This aligns with findings from other PPAs in pain management trials. The PPA for CARES trial, which examined willingness to participate in a study comparing opioid-based and opioid-sparing postoperative analgesic regimens, reported a willingness rate of 71%.^23^ Similarly, the PPA for PEACE-PAIN trial, which assessed attitudes toward psilocybin for chronic neuropathic pain, found that 76.9% of the participants were willing to enroll.^32^ Our slightly higher willingness rate may be attributed to the strong association between pain as a major surgical concern and trial participation willingness, with 88% of participants citing pain management as a primary motivator. This trend is consistent with prior PPAs, where dissatisfaction with current pain management and the potential for improved analgesia were key enrollment drivers. This underscores the importance of addressing patient-specific clinical needs and suggests that targeted messaging focused on the potential for pain relief and improved recovery outcomes may be particularly effective in recruitment efforts.

Remarkably, spinal fusion surgery is frequently associated with chronic pain and poorly managed postoperative pain,^1,2,33^ highlighting the persistent unmet need for effective postoperative analgesia in spine surgery. As such, the promise of a novel approach like rectal methadone appears to resonate strongly. Additional factors, including perceived benefits of rectal methadone, altruism, and contributing to scientific advancement, were common motivating themes, reflecting both personal and societal drivers. Likewise, previous studies showed that participants are often motivated by a combination of self-interest and desire to advance healthcare knowledge.^34,35^ These findings highlight the importance of framing trial participation as a dual opportunity for personal and collective benefit.

Barriers to participation were primarily potential side effects, fear of dependency or addiction, and general skepticism toward analgesics. These concerns, aligned with previous studies on apprehensions surrounding opioid-based treatments,^19,20^ were consistent across both willing and not willing groups, albeit more pronounced among those unwilling to participate. Nevertheless, despite mention of unique concerns, most participants remained willing to participate in the PROMISE trial, suggesting that concerns can often coexist with willingness to participate if addressed effectively. Notably, while rectal administration was initially perceived as a novel route, most participants viewed it positively, understanding its pharmacokinetic advantages and potential for better postoperative pain control. The general positive receptivity to rectal administration suggests that this route, despite being underutilized in clinical pain management, holds promise for broader application.^36–38^ Interestingly, several participants who expressed skepticism about conventional opioids were still willing to consider rectal methadone. This presents an opportunity to frame rectal methadone not merely as another opioid but as an effective alternative to intravenous methadone, especially in regions where intravenous administration is not available. Ultimately, this will help us design our study messaging to more effectively align with the specific needs of this patient population.

### Addressing barriers to willingness to participate through clinical trial design

Key concerns identified in this PPA study can inform refinements to the PROMISE trial protocol to enhance patient recruitment and adherence. Comprehensive pre-trial education is essential to address:

1. The safety profile of methadone, including emphasizing its lower risk of dependency, compared to conventional opioids, with safeguards in the controlled perioperative context.
2. The pharmacological benefits of the rectal route, particularly its consistent absorption and sustained analgesic effects.
3. Clarifications about trial design, including randomization, blinding, and availability of
4. rescue medications.
5. Detailed information about potential side effects and clarify that robust safety measures are in place, including frequent monitoring for adverse effects during and after surgery.

Such targeted and feasible modifications may help alleviate concerns, improve participant enrollment and retention in the PROMISE trial, and inform general patient-informed adaptations for future clinical trials (Figure 4).

**Figure 4.**
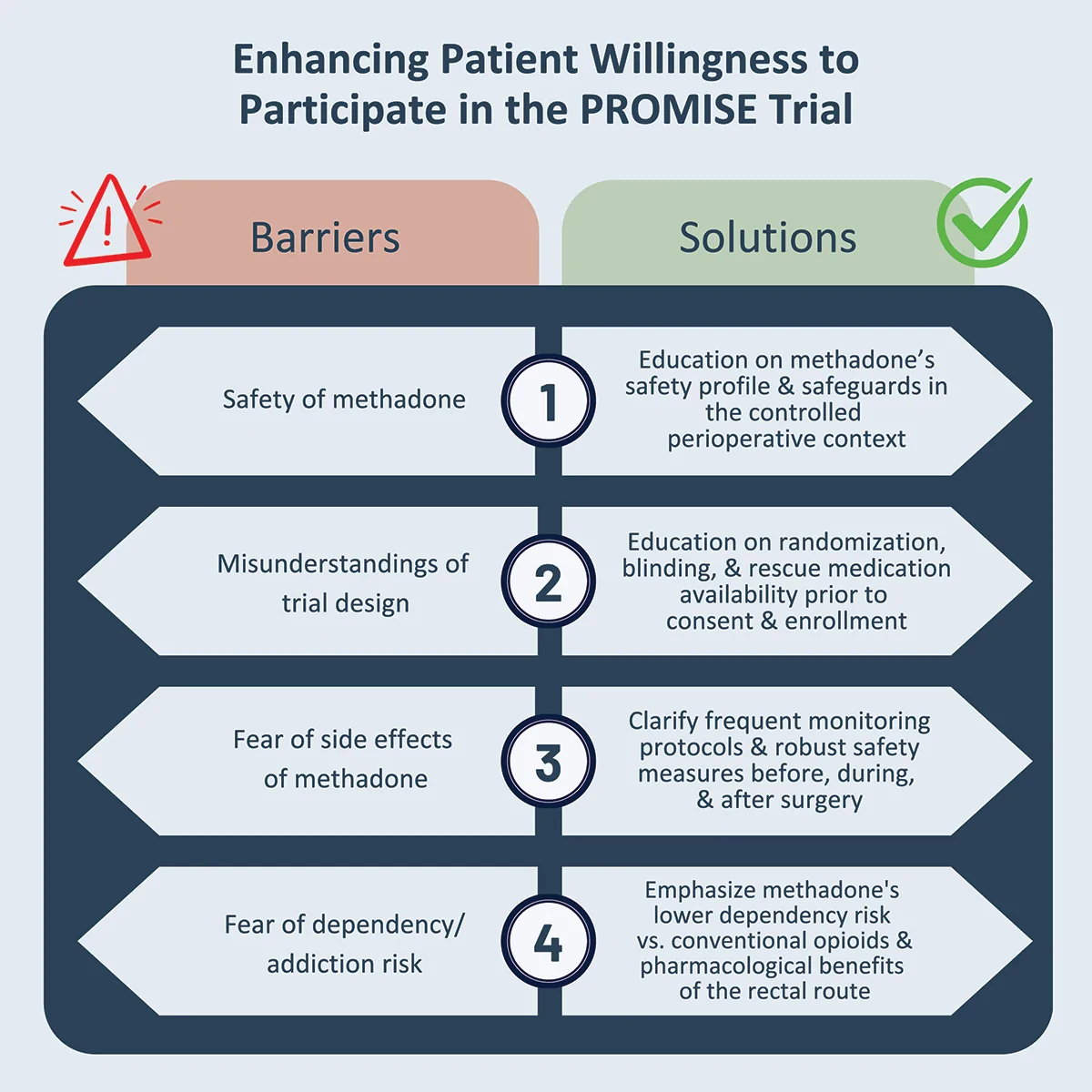
An infographic outlining key barriers to patient enrollment and the associated solutions to enhance patient willingness to participate in the PROMISE Trial.

### Strengths, limitations, and future directions

A key strength of this study lies in its comprehensive mixed-methods design, which provided both quantitative and qualitative insights into factors influencing trial participation. Thematic saturation in qualitative interviews further supports the robustness of findings and ensures that key motivators and barriers to participation were adequately captured.^39^

However, several limitations warrant consideration. The single-center design and small sample size may limit generalizability. Since intravenous methadone is available in some jurisdictions but not others, the findings will be most applicable to settings where intravenous methadone is unavailable. Given the exploratory nature and modest, unbalanced subgroup sizes of this assessment, between-group comparisons were regarded as hypothesis-generating and are reported alongside the full descriptive distribution of Likert responses. Future studies should explore multi-center recruitment and include diverse populations to validate and expand on these findings. Another limitation pertains to the specificity of the information provided to participants about the intervention. The Letter of Information explicitly referenced rectal methadone, which may have inadvertently introduced bias, particularly among individuals with strong preconceived opinions about opioids or the rectal route of administration. This likely biased our results toward a sample that was more willing to participate, as those with negative perceptions of opioids or rectal administration may have self-selected out of participating. A more generalized description, such as, “a trial investigating a new method for pain management after spine surgery,” could have reduced selective nonparticipation and yielded a more representative sample. This adjustment may enhance the validity and generalizability of future preference assessments, particularly in measuring willingness to participate in trials involving novel interventions. Further, while the PPA provides valuable insights into factors influencing willingness to participate, it does not guarantee that all willing participants will follow through with enrollment in the actual trial. Future research should examine factors influencing retention and attrition once the trial begins. To mitigate potential dropout, strategies, such as personalized follow-up, clear communication of study expectations, and participant-centered support should be considered. Additionally, assessing whether participants who initially express willingness encounter barriers during the trial will provide further insights into optimizing recruitment and retention efforts in perioperative pain trials. While our sample was predominantly White/Caucasian (80%), our recruitment efforts were limited to patients undergoing elective spine surgery at a single academic hospital, with the exclusion of non-English-speaking patients, which may have influenced the demographic composition. Future studies should consider multilingual recruitment materials and interpreter services to enhance accessibility and representation.

Finally, while this study focused on patient willingness, understanding the long-term impact of rectal methadone on postoperative recovery and opioid dependency remains a critical area for future research. Investigating these outcomes in a large-scale RCT could establish rectal methadone as a cornerstone of perioperative pain management strategies, advancing care for spine surgery patients and informing best practices in clinical trial design.

## Conclusion

Overall, this PPA demonstrated that 83.3% of the potential participants were willing to participate in the PROMISE trial. We identified key motivating themes and concerns for patient participation that should improve recruitment and impact the PROMISE trial and that may also be generalizable to other postoperative pain studies. Our findings contribute to a growing body of evidence supporting innovative approaches to perioperative pain management, with the potential to improve patient-important outcomes through patient-centered trial designs.^40^

## Appendices

### Appendix I

PPA administered to each patient consisting of (1) a vignette describing the proposed trial; (2) an assessment of the patients’ understanding of the proposed trial; (3) open-ended questions assessing patients’ perceptions and attitudes toward the trial; (4) patient-completed questionnaire.

### A. PPA semi-structured interview

Script for interviewer:

Thank you for agreeing to participate in our survey. This survey is entirely voluntary. I will describe the new study and tape your answers, then I will provide a questionnaire for you to respond. The survey will take about 15 minutes. Direct quotes may be used in the presentation of the results, but no names will be attached to the quotes. You may skip or not answer any question you do not wish to answer, and you can pause or stop the interview for any reason.

First, I am going to read to you the description about the study we are planning. Then, I will start recording your responses to a 2 sets of questions. (1) I will ask you about your understanding of the proposed study and (2) I will ask you about your opinion on factors that might motivate or concern you about participating in the study we are planning.

### Description of PROMISE Study (to be read to each patient)

Researchers at St. Michael’s Hospital are studying how to best manage pain after spine surgery. One way to help with pain after surgery is by giving pain medication ***before*** surgery when you’re put to sleep with general anesthesia. Since currently the best ***type*** of medication to give to help manage pain after spine surgery is not known, we are looking to find patients such as yourself to participate in a study examining the most effective way to treat perioperative pain. Other studies have shown that methadone, which is a type of opioid, helps decrease the pain patients feel right after surgery, reduces how much strong pain medicine patients may need, and lowers the chances of pain becoming a long-term problem after surgery. Particularly rectal methadone (giving the medication through the rectum) is absorbed into the body in a way that makes it longer-lasting and potentially better suited for spine surgery.

I understand the thought of a rectal medication can sound quite strange; but given its potential benefits, we aim to help patients feel more comfortable with this route of administration by giving it once they are under general anesthesia – so they won’t feel a thing! In this study, participants will either receive a single dose of rectal methadone or a single dose of rectal saline solution, which is just a saltwater placebo. Participants will be assigned to one of the two interventions at random, like flipping a coin. All patients and staff will be unaware of the intervention assignment, except for the anesthesia provider to make sure the patient is safe in all cases. To make sure everyone gets adequate treatment for their pain, all participants will receive the usual care for spine surgery and pain relief as decided by the doctors taking care of them.

Someone from the research team will assess patients on the first, second, and third days after surgery, and then again by phone 30 days after surgery to see how well controlled their pain is.

### Questions assessing patients’ understanding of the trial:

1. Can you tell me what the two intervention options are to which participants can be assigned?
2. Can you tell me whether patients in the trial will be able to choose or know if they receive the rectal methadone or not?
3. How and when will research staff contact study participants follow-up once they leave the hospital?

### Open-ended questions about factors influencing enrollment decisions:

1. What factors might motivate you to participate in this study?
2. What concerns do you have about participating in this study?

### B. PPA questionnaire

*Questionnaire (to be completed by each patient*):

1. Considering this information, how willing would you be to participate in this study if it were to happen in the future? Please circle your response on this scale from 1 to 6, where 1 means you would **definitely not participate**, and 6 means you **definitely would participate**.
  **Table:**
  1	2	3	4	5	6
  Definitely Not	Probably Not	Maybe Not	Maybe	Probably	Definitely
2. What year were you born? ___________
3. Sex assigned at birth:
  □ Male □ Female □ Intersex □ Prefer not to answer □ Prefer to self-describe **___________**
4. Do you consider yourself to be a visible minority?
  □ Yes □ No
5. How many years of school have you completed? Please circle one number.
  **Table:**
  1 2 3 4 5 6 7 8	9 10 11 12	13 14 15 16	17 18 19 20+
  Grade School	High School	Post-Secondary	Graduate School
6. Have you ever participated in a research study before?
  □ Yes □ No □ Not Sure
  If yes, what did you do?
  □ I started the study but later withdrew □ I completed the study □ The study is still on-going (I’m actively participating)
7. Do you currently have any concomitant medical conditions, other than the reason you are receiving spinal surgery?
  □ Yes □ No
8. Have you ever had surgery before?
  □ Yes □ No
9. Are you currently taking any medication for pain regularly (either prescription or over the counter)?
  □ Yes □ No
10. Is pain one of the major concerns you have regarding your surgery?
  □ Yes □ No
11. Has anyone discussed how to manage your pain after your surgery?
  □ Yes □ No
12. Over the past 3 months have you had significant pain on most days?
  □ Yes □ No
  If yes, do you feel as though you are managing your pain well?
  □ Yes □ No
13. Over the past month have you used any medication for pain for more than a week?
  □ Yes □ No
14. Over the past month have you used anything other than medication to manage your pain for more than a week? (ex. Heat pack, warm bath, massage, alcohol use, etc.)
  □ Yes □ No

## Appendix II

### Frequencies of motivating and concerning factors identified from patient interviews influencing willingness to participate in the PPA-PROMISE trial

*Data presented as N (%) or mean ± SD*

**Table:**

	Total (n = 30), N (%) or mean (SD)	Not willing to participate (n = 5, 16.7%), N (%) or mean (SD)	Willing to participate (n = 25, 83.3%), N (%) or mean (SD)
**Identified Themes: Motivators**			
Optimizing pain management/pain relief	17 (57)	1 (20)	16 (64)
Perceived comfort/benefit of rectal route of administration	6 (20)	1 (20)	5 (20)
Perceived benefit of rectal methadone	9 (30)	1 (20)	8 (32)
Curiosity	6 (20)	0 (0)	6 (24)
Familiarity with methadone	2 (7)	0 (0)	2 (8)
Scientific/knowledge advancement	7 (23)	0 (0)	7 (28)
Altruism	8 (27)	0 (0)	8 (32)
Minimizing post-operative opioid use	3 (10)	0 (0)	3 (12)
Ambivalence	1 (3)	0 (0)	1 (4)
No motivating factors	4 (13)	3 (60)	1 (4)
**Identified Themes: Concerns**			
Concerns about long-term methadone use and effectiveness	1 (3)	0 (0)	1 (4)
Safety of methadone	1 (3)	0 (0)	1 (4)
Side effects of methadone	4 (13)	1 (20)	3 (12)
Opposition to receiving placebo	1 (3)	0 (0)	1 (4)
Stigma around methadone	2 (7)	0 (0)	2 (8)
Influence on perception of pain	1 (3)	0 (0)	1 (4)
Impact on post-surgical pain management	1 (3)	0 (0)	1 (4)
Impact on surgical performance, outcomes and/or timeline	2 (7)	0 (0)	2 (8)
Skepticism/hesitance toward analgesics in general	3 (10)	2 (40)	1 (4)
Fear of potential for dependency or addiction	4 (13)	2 (40)	2 (8)
Blinding to intervention assignment	1 (3)	0 (0)	1 (4)
Novelty of study	2 (7)	0 (0)	2 (8)
Lack of education/knowledge regarding study drugs	3 (10)	1 (20)	2 (8)
Ambivalence	1 (3)	0 (0)	1 (4)
No concerns	14 (47)	1 (20)	13 (52)
